# Complete Genome Sequence of the N4-like Pseudomonas aeruginosa Bacteriophage vB_PaeP_CMS1

**DOI:** 10.1128/mra.00239-22

**Published:** 2022-05-31

**Authors:** Dominick Faith, Margie Kinnersley, Caleb M. Schwartzkopf, Camilla D. de Mattos, Amelia K. Schmidt, Patrick R. Secor

**Affiliations:** a Division of Biological Sciences, University of Montana, Missoula, Montana, USA; Queens College CUNY

## Abstract

vB_PaeP_CMS1 is a lytic bacteriophage that infects Pseudomonas aeruginosa. Whole-genome sequencing revealed that vB_PaeP_CMS1 has a linear double-stranded DNA (dsDNA) genome composed of 72,673 bp, with a GC content of 54.9%, that harbors 92 predicted open reading frames, with the greatest genome homology to members of the family *Podoviridae*, genus *Litunavirus*.

## ANNOUNCEMENT

We recently reported that bacteriophage vB_PaeP_CMS1 does not utilize type IV pili as a receptor during infection of Pseudomonas aeruginosa (1). Here, we announce the genome sequence of the vB_PaeP_CMS1 bacteriophage.

Bacteriophage vB_PaeP_CMS1 was originally isolated from an uncharacterized phage cocktail sent to us by the Bollyky laboratory at Stanford University. Pseudomonas aeruginosa strain PAO1 Δ*pilA* was used as a host. Phages in the cocktail were propagated with PAO1 Δ*pilA* at 37°C in lysogeny broth (LB). Soft-agar overlays (2) were used to isolate individual plaques, followed by propagation in liquid cultures (3). Filtered vB_PaeP_CMS1 lysates were then treated with 20 mg/mL DNase and 20 mg/mL RNase. To denature phage virions, vB_PaeP_CMS1 lysates were treated with 100 μg/mL proteinase K, 0.5% sodium dodecyl sulfate (SDS), 100 mM EDTA, and 10% cetyltrimethylammonium bromide (CTAB) with 0.7 M NaCl. DNA was extracted using phenol/chloroform/isoamyl alcohol (25:24:1), followed by ethanol precipitation and resuspension in 10 mM Tris-HCl (pH 8.5). vB_PaeP_CMS1 DNA concentration and purity were then estimated with a NanoDrop spectrophotometer.

**FIG 1:**
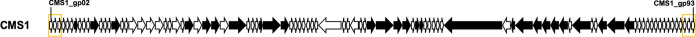
Pseudomonas aeruginosa bacteriophage vB_PaeP_CMS1 annotated genome. The 72,698-bp genome of bacteriophage vB_PaeP_CMS1 is predicted to harbor 92 ORFs (vB_PaeP_CMS1_gp02-vB_PaeP_CMS1_gp93), which are represented by arrows. The 619-bp terminal repeats are indicated with orange boxes, and ORFs harbored by all N4-like phages sequenced thus far are colored black.

Purified vB_PaeP_CMS1 DNA was sent to the Microbial Genome Sequencing Center (MiGS) for whole-genome sequencing (200-Mbp package). Sample libraries were prepared using the Illumina DNA preparation kit and IDT 10-bp unique dual indices (UDIs) and sequenced on an Illumina NextSeq 2000 system, producing 1,020,000 reads (2 × 151 bp). Demultiplexing, quality control, and adapter trimming were performed with bcl-convert (v3.9.3). The paired-end reads were processed using the Geneious Prime software platform (v2022.1.1). The reads were trimmed using BBduk (v38.46, with default parameters) and normalized using BBNorm (v38.46, with default parameters). Trimmed and normalized reads were then assembled using the Geneious Prime *de novo* assembler (with default parameters); 27,493 trimmed and normalized reads were assembled into a single contig consisting of 72,698 bp with a mean coverage of 55.9 reads. Genomic homologs were identified with BLASTn (4). Open reading frames (ORFs) were predicted using the RAST platform (5), followed by manual analysis with BLASTp (4), HHPRED (6), and Pfam (7). The vB_PaeP_CMS1 genome contains 619-bp terminal direct repeats (Fig. 1).

These analyses indicate that vB_PaeP_CMS1 is an obligate lytic N4-like bacteriophage that shares significant sequence homology (BLASTn) with phages belonging to the family *Podoviridae*, genus *Litunavirus.* vB_PaeP_CMS1 virions package a 72,673-bp linear double-stranded DNA (dsDNA) genome with a GC content of 54.9% and 92 predicted ORFs. At the DNA level, vB_PaeP_CMS1 shares the greatest sequence homology with phage vB_PaeP_PYO2 (99.73%) (8). Additionally, vB_PaeP_CMS1 shares 92/96 ORFs with the previously characterized phage PEV2 (9).

### Data availability.

The genome sequence and associated data for phage vB_PaeP_CMS1 were deposited under GenBank accession number OM937766, BioProject accession number PRJNA811632, SRA accession number SRR18189815, and BioSample accession number SAMN26345544.
